# Vitamin K1 inhibits ferroptosis and counteracts a detrimental effect of phenprocoumon in experimental acute kidney injury

**DOI:** 10.1007/s00018-022-04416-w

**Published:** 2022-06-28

**Authors:** Benedikt Kolbrink, Friedrich Alexander von Samson-Himmelstjerna, Maja Lucia Messtorff, Theresa Riebeling, Raphael Nische, Jessica Schmitz, Jan Hinrich Bräsen, Ulrich Kunzendorf, Stefan Krautwald

**Affiliations:** 1 Department of Nephrology and Hypertension, University Hospital Schleswig-Holstein, Campus Kiel, Fleckenstr. 4, 24105 Kiel, Germany; 2 Nephropathology Unit, Institute of Pathology, University of Hannover, 30625 Hannover, Germany

**Keywords:** Vitamin K, Ferroptosis, Acute kidney injury, Ischemia–reperfusion injury

## Abstract

Ferroptosis, a type of iron-dependent programmed cell death distinct from apoptosis, necroptosis, and other types of cell death, is characterized by lipid peroxidation, reactive oxygen species production, and mitochondrial dysfunction. Accumulating evidence has highlighted vital roles for ferroptosis in multiple diseases, including acute kidney injury. Therefore, ferroptosis has become a major focus for translational research. However, despite its involvement in pathological conditions, there are no pharmacologic inhibitors of ferroptosis in clinical use. In the context of drug repurposing, a strategy for identifying new uses for approved drugs outside the original medical application, we discovered that vitamin K1 is an efficient inhibitor of ferroptosis. Our findings are strengthened by the fact that the vitamin K antagonist phenprocoumon significantly exacerbated ferroptotic cell death in vitro and also massively worsened the course of acute kidney injury in vivo, which is of utmost clinical importance. We therefore assign vitamin K1 a novel role in preventing ferroptotic cell death in acute tubular necrosis during acute kidney injury. Since the safety, tolerability, pharmacokinetics, and pharmacodynamics of vitamin K1 formulations are well documented, this drug is primed for clinical application, and provides a new strategy for pharmacological control of ferroptosis and diseases associated with this mode of cell death.

**Supplementary Information:**

The online version contains supplementary material available at 10.1007/s00018-022-04416-w.

## Introduction

Acute kidney injury (AKI) occurs in up to 20% of hospitalized patients and is a major clinical problem that is associated with high mortality and morbidity [1]. Therapy for AKI currently consists of supportive measures in addition to removal of trigger factors [2]. Despite major scientific efforts, there is currently no established therapy that can halt pathological cell death in the kidney, as it occurs during acute tubular necrosis in AKI. In recent years, it has become evident that the cell death form ferroptosis is the most pathophysiologically relevant form of cell death in acute tubular necrosis during AKI [3–5]. First recognized as a distinct entity in 2008 [6], and named in 2012 [7], ferroptosis is a form of inflammatory cell death, characterized by excessive iron-dependent lipid peroxidation in the plasma membrane [8, 9]. Three major enzymatic protective mechanisms have been described that guard cells against this lethal accumulation of lipid peroxides: (1) cytosolic glutathione peroxidase 4 (GPX4) catalyzes the reduction of lipid peroxides in a glutathione (GSH)-dependent manner, which relies on cellular cystine import via the system x_c_^−^ transporter [3, 7, 10]; (2) ferroptosis suppressor protein 1 (FSP1), an oxidoreductase of the plasma membrane dependent on coenzyme Q10 [11, 12]; and (3) dihydroorotate dehydrogenase (DHODH), which mainly reduces lipid peroxides in mitochondria [13]. If these protective mechanisms are inactivated by pharmacological agents or in the context of pathological conditions, ferroptosis occurs, as is the case in AKI. Once activated, ferroptosis spreads to neighboring cells due to its propagative nature [14, 15], leading to pronounced kidney cell death and organ failure [3–5].

Ferroptosis can be efficiently inhibited by specific antioxidant substances active in lipid membranes [16, 17], although no substance is yet in clinical use as a ferroptosis inhibitor. Therefore, the identification of a safe, stable, and clinically applicable ferroptosis inhibitor for use in humans is an attractive therapeutic strategy for the treatment of AKI. A promising candidate in this respect is vitamin K1, a fat-soluble vitamin that has been used safely and reliably for decades in the prophylaxis of vitamin K deficiency bleeding in neonates [18, 19] and for various ailments in adults, without showing toxicity even at very high doses [20–22].

Already in the last millennium, several groups described that vitamin K and its derivatives can prevent lipid peroxidation and may even be more potent for this purpose than vitamin E (tocopherol) [23–26], which is widely regarded as the most important antioxidant protective mechanism of biomembranes [27]. Furthermore, reports from the early 2000s showed that vitamin K can inhibit glutathione depletion-mediated and lipoxygenase-dependent oxidative cell death in neurons and oligodendrocytes [28, 29], a type of cell death that shares many common features with ferroptosis. Based on this, we hypothesized that vitamin K, which is already in wide clinical use for other indications, might also be a potent inhibitor of ferroptosis, and it could potentially be safely used to treat AKI in humans.

## Materials and methods

### Cell lines

NIH3T3 and HT-1080 cells were obtained from American Type Culture Collection (Manassas, VA, USA). Both cell lines were cultured in Dulbecco’s modified Eagle’s medium (DMEM, Gibco, Thermo Fisher Scientific, Schwerte, Germany) supplemented with 10% (vol/vol) fetal calf serum (FCS, PAN-Biotech GmbH, Aidenbach, Germany), 100 U/ml penicillin, and 100 μg/ml streptomycin (Merck Millipore GmbH, Darmstadt, Germany). For HT-1080 cells, the medium was additionally supplemented with MEM NEAA (Gibco, Thermo Fisher Scientific).

Murine proximal tubular epithelial cells (MCTs) were a generous gift from Alberto Ortiz (Department of Medicine, Universidad Autonoma de Madrid, Madrid, Spain). These cells were cultured in Roswell Park Memorial Institute (RPMI) 1640 medium (Gibco, Thermo Fisher Scientific) supplemented with 10% (vol/vol) FCS, 100 U/ml penicillin, and 100 μg/ml streptomycin.

All cell lines were cultured in a humidified 5% CO_2_ atmosphere at 37 °C. Negativity for mycoplasma was routinely checked using a MycoAlert™ Mycoplasma Detection Kit (Lonza, Cologne, Germany). Unless stated otherwise, assays were conducted on 1 × 10^5^ cells in 1 ml of medium.

Mouse embryonic fibroblasts (MEFs) from C57BL/6 J mice were generated in our lab from E13.5 embryos as described previously [30]. Briefly, after the placenta, yolk sac, head, and dark red organs had been removed, embryos were finely minced and digested for 20 min in 0.25% trypsin. A single-cell suspension was seeded for culture. Primary MEFs were cultured in DMEM supplemented with 10% FCS, 50 U/ml penicillin, 50 μg/ml streptomycin, and 7 µl/l β-mercaptoethanol.

Primary murine proximal tubular cells (PTCs) were isolated as described previously [31]. Briefly, 6-week-old C57BL/6 J mice were sacrificed, their kidneys were removed, and the cortices were harvested. Cortex tissue was chopped and placed into Hank’s balanced salt solution (HBSS, Gibco, Thermo Fisher Scientific), supplemented with 15 mM glucose, 1 mM l-alanine, 5 mM glycine and 15 mM HEPES, to which 0.1% collagenase type 2 (Sigma-Aldrich, Taufkirchen, Germany) and 96 µg/ml soybean trypsin inhibitor (Sigma-Aldrich) were added beforehand. Minced cortex tissue was incubated for 30 min at 37 °C. Digested cortices were transferred through a 250 µm sieve (Thermo Fisher Scientific), briefly centrifuged (170×*g* at room temperature), resuspended, and passed through a 100 µm nylon cell strainer (Corning Incorporated, NY, USA via Merck). Tubule fragments were collected from the sieve using the HBSS solution mentioned above, supplemented with 1% bovine serum albumin. After centrifugation (170×*g,* 5 min at room temperature), the pellet was resuspended in medium consisting of phenol red free 1:1 DMEM/F12 supplemented with a mixture of 1 × ITS (Gibco, Thermo Fisher Scientific), 50 nM hydrocortisone (Sigma-Aldrich), MEM NEAAs, 1% (v/v) FCS, 100 U/ml penicillin and 100 μg/ml streptomycin. Tubule fragments were plated on 24-well plates (Cell+, Sarstedt, Nümbrecht, Germany) and cultured at 37 °C in a humidified 5% CO_2_ atmosphere. Over 90% purity of proximal tubular fragments was ensured by fluorescence microscopy after staining for the proximal tubular marker megalin [32] (antibody sc-515750, Santa Cruz Biotechnology, Heidelberg, Germany). Within 1 week, cells grew from the tubule fragments, reached 70% confluency and were used for experiments.

### Reagents

Erastin (571203-78-6) and RSL3 (1219810-16-8) were bought from Tocris Bioscience (Bio-Techne GmbH, Wiesbaden, Germany). Ferrostatin-1 (SML0583), phenprocoumon (SML2365) and iFSP1 (SML2749) were purchased from Sigma-Aldrich. Brequinar (HY-108325) was purchased from MedChemExpress (via Hölzel Diagnostika, Cologne, Germany). Vitamin K1 (phytomenadione) dissolved at a concentration of 10 mg/ml in glycocholic acid, [3-sn-phosphatidyl]choline, and sodium hydroxide was obtained from CHELAPHARM Arzneimittel GmbH (Greifswald, Germany).

### Flow cytometry analysis of cell death

Phosphatidylserine exposure to the outer cell membrane of apoptotic cells or inner plasma membrane of necrotic cells and incorporation of 7-amino-actinomycin D (7-AAD) into necrotic cells were quantified by flow cytometry analysis. After stimulation of cells under the indicated conditions, staining was performed on single-cell suspensions using FITC Annexin V (BioLegend, 640906) and 7-AAD Viability Staining Solution (BioLegend, 420404) according to the manufacturer’s instructions. Fluorescence was analyzed using an FC-500 flow cytometer (Beckman Coulter GmbH, Krefeld, Germany).

### Analysis of cellular lipid peroxide generation

Cells were treated for 4 h with 5 µM RSL3 (± 1 µM ferrostatin-1 or 10 µM vitamin K1, respectively) and harvested by trypsinization. Thereafter, the cells were suspended in 500 µl PBS containing 2 µM BODIPY® (581/591) C11 (Gibco, Thermo Fisher Scientific). Afterwards, cells were incubated for 10 min at 37 °C in a tissue culture incubator, centrifuged (400 × *g*, 10 min, 4 °C), resuspended in 700 µl of fresh PBS, passed through a 40 µM cell strainer (BD Biosciences, Heidelberg, Germany), and analyzed using a FC-500 flow cytometer equipped with a 488 nm laser for excitation. Data were collected from the FL1 channel, and a minimum of 10,000 cells were analyzed per condition.

### Analysis of cell death by western blotting

For immunoblotting, 1 × 10^5^ adherent cells were seeded in 6-well plates and 24 h later treated as indicated. Thereafter, the cells were harvested, washed, and lysed in ice-cold modified Frackelton buffer (10 mM Tris–HCl (pH 7.5), 50 mM NaCl, 1% Triton X-100, 30 mM Na_4_P_2_O_7_, 50 mM NaF, 100 μM Na_3_VO_4_, 2 μM ZnCl_2_), containing 1 mM C_7_H_7_FO_2_S (PMSF). Similarly, the kidney lysates used to prepare immunoblots shown in Fig. 5d were obtained by homogenization in this buffer. Insoluble material was removed by centrifugation (14,000×*g*, 10 min, 4 °C), and protein concentrations were quantified using a commercial Bradford assay kit (Bio-Rad GmbH, Munich, Germany) according to the manufacturer’s instructions. Equal amounts of protein (20 μg per lane) were resolved by reducing SDS-PAGE and transferred to a polyvinylidene fluoride (PVDF) membrane (GE Healthcare Life Sciences, Freiburg, Germany). Membranes were probed with specific primary antibodies against acyl-CoA synthetase long-chain family member 4 (ACSL4; ab155282, Abcam, Berlin, Germany) or GPX4 (ab125066; Abcam), respectively, and corresponding secondary horseradish peroxidase (HRP)-conjugated polyclonal goat anti-rabbit immunoglobulin (Jackson ImmunoResearch Laboratories, Inc., West Baltimore Pike, PA, USA; #111-035-003 via Dianova, Hamburg, Germany).

Autoradiographs were generated using Amersham Hyperfilm MP high-performance autoradiography films (GE Healthcare 28906842) and developed with a Curix 60 X-ray film processor (AGFA, Mortsel, Belgium). To re-probe the same membrane, the membrane was stripped using a commercial stripping buffer (Gibco, Thermo Fisher Scientific) before incubation with anti-β-actin antibody (#4967; Cell Signaling Technology, Frankfurt, Germany).

### Mice

All mice used in our in vivo studies were 8-week-old males of the C57BL/6 J background. Animals were purchased from Janvier Labs (Saint Berthevin, France), and housed in the Central Animal Facility of the University Hospital Schleswig–Holstein (Kiel, Germany). They received standard chow and water ad libitum and were maintained under a 12 h day–night cycle. All in vivo experiments were conducted in accordance with the animal protection regulations of the local authorities and were approved by the Ministry of Energy, Agriculture, the Environment, Nature and Digitalization of Schleswig–Holstein, Germany.

To isolate PTCs, rodents were first anesthetized with isoflurane followed by euthanasia through cervical dislocation. All experiments were performed according to the Protection of Animals Act with the approval of German authorities.

### Renal ischemia–reperfusion injury (IRI)

Induction of murine renal IRI was performed as described previously [30]. Kidneys were exposed via a midline abdominal incision and bilateral renal pedicle clamping for 35 min using microaneurysm clamps (Aesculap Inc., Center Valley, PA, USA). Throughout the surgical procedure, the mice were kept under isoflurane narcosis, and their body temperature was maintained at 36–37 °C by continuous monitoring using a temperature-controlled, self-regulated heating system (Fine Science Tools, Heidelberg, Germany). After clamps were removed, kidney reperfusion was confirmed visually before the abdomen was closed in two layers using standard 6-0 sutures. To maintain fluid balance, all mice were supplemented with 1 ml of prewarmed PBS administered intraperitoneally directly after surgery. After 48 h of reperfusion, the mice were sacrificed, blood samples were obtained by retrobulbar puncture, and kidneys were collected for analysis.

### Plasma parameters

Using a heparinized capillary tube, whole blood was collected from the retrobulbar capillary bed and plasma was obtained by centrifugation. Creatinine and urea concentrations were measured photometrically at the Central Laboratory of the University Hospital Schleswig–Holstein Kiel, Germany.

### Histology

Kidneys were fixed in 4% neutral-buffered formaldehyde and embedded in paraffin. The 3 μm sections produced were dewaxed, rehydrated, and subjected to periodic acid-Schiff staining according to routine protocols, and evaluated blinded by an experienced nephropathologist.

Antigen retrieval for ACSL4 was performed in 0.01 M sodium citrate buffer (pH 6.0), for 30 min at 98 °C followed by 10 min in 3% H_2_O_2_ to block endogenous peroxidase activity. Sections were incubated for 60 min with a 1:200 dilution of anti-ACSL4 antibody ab155282 (Abcam). For ACSL4 detection, HRP-conjugated polyclonal donkey anti-rabbit immunoglobulins (Jackson ImmunoResearch Laboratories; 711-035-152 via Dianova) were used at a 1:100 dilution as secondary antibody for 30 min followed by 3,3’-diaminobenzidine detection. Subsequently, sections were mildly counterstained with hemalum. Sections were evaluated using an Olympus U-DO3 microscope, and representative photomicrographs were taken using a Jenoptic ProgRes® SpeedXT core 5 (Jena, Germany).

### Statistical methods and analyses

For all experiments, differences between datasets were considered statistically significant when *p* values were < 0.05, if not otherwise specified. Statistical comparisons were performed using the two-tailed Student’s *t* test for comparisons between two groups. Comparisons between multiple groups were performed by a one-way analysis of variance with Bonferronis’s post hoc test. Asterisks are used in the figures to specify statistical significance (*p < 0.05; **p < 0.01; ***p < 0.001). Results are presented as means ± standard deviation (SD) unless otherwise specified.

## Results

### Vitamin K1 inhibits ferroptosis in vitro

**Fig. 1:**
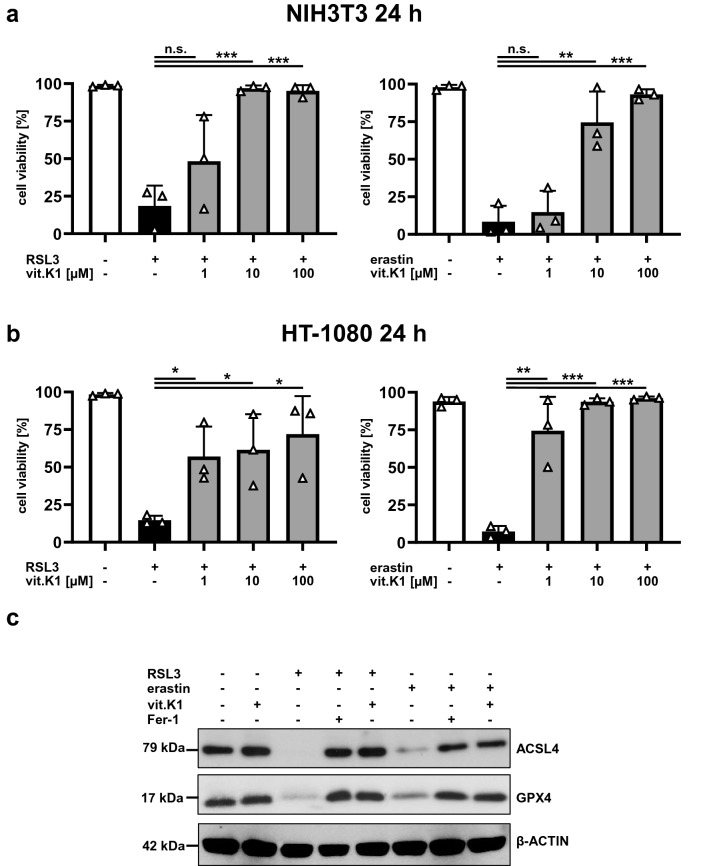
Vitamin K1 inhibits ferroptosis in vitro. **a** Murine NIH3T3 cells were pretreated at 37 °C for 30 min in the absence or presence of increasing (indicated) concentrations of vitamin K1 (vit.K1). Ferroptosis was induced thereafter for 24 h by the addition of 5 µM RSL3 or 2.5 µM erastin, respectively. **b** Human HT-1080 cells were pretreated at 37 °C for 30 min in the absence or presence of increasing (indicated) concentrations of vitamin K1. Ferroptosis was induced thereafter for 24 h by the addition of 5 µM RSL3 or 25 µM erastin, respectively. Cell death was quantified by FACS analysis using 7-amino-actinomycin D (7-AAD) and phosphatidylserine accessibility (Annexin V staining) as markers. Each graph shows the mean ± SD of three independent experiments. **c** NIH3T3 cells were pretreated at 37 °C for 30 min in the presence or absence of 1 µM ferrostatin-1 (Fer-1) or 10 µM vitamin K1, as indicated. Ferroptosis was induced thereafter for 24 h by addition of 5 µM RSL3 or 2.5 µM erastin. Equal amounts of protein (20 µg/lane) were resolved by SDS-PAGE, and expression of ACSL4 was detected by western blotting. The blot was stripped and re-probed with an antibody against GPX4 and thereafter β-actin as a loading control

Although the antioxidant characteristics of vitamin K are often neglected when its functional role is discussed [33], it can inhibit lipid peroxidation and glutathione depletion-mediated oxidative cell death [26, 29]. Therefore, we explored the potential inhibitory effect of vitamin K1 on ferroptosis in vitro. To this end, we induced ferroptosis in murine (NIH3T3 fibroblasts) and human (HT-1080 fibrosarcoma) transformed cell lines by incubating with the canonical ferroptosis inducers RSL3 (GPX4 inhibitor) [6, 10] or erastin (system x_c_^−^ inhibitor) [7, 34]. Incubation with RSL3 or erastin for 24 h resulted in pronounced cell death in all cell lines studied, and this could be inhibited in a dose-dependent manner by vitamin K1 (Fig. 1a and b). Incubation with vitamin K1 alone at doses of up to 100 µM had no effect on the survival of the cell lines studied after 24 h (Supplemental Fig. 1). As we showed previously [5], ACSL4, a crucial contributor to ferroptosis that provides the plasma membrane with oxidation-sensitive polyunsaturated ω6 fatty acids [35–37], is degraded in vitro throughout the course of ferroptosis. This time-dependent process (Supplemental Fig. 2) which is accompanied by the simultaneous depletion of GPX4 protein (Fig. 1c), correlates with the degree of ferroptotic cell death, and can be prevented efficiently by vitamin K1 (Fig. 1 and Supplemental Fig. 2).

### The inhibitory properties of vitamin K1 during ferroptosis are cross-cellular

**Fig. 2:**
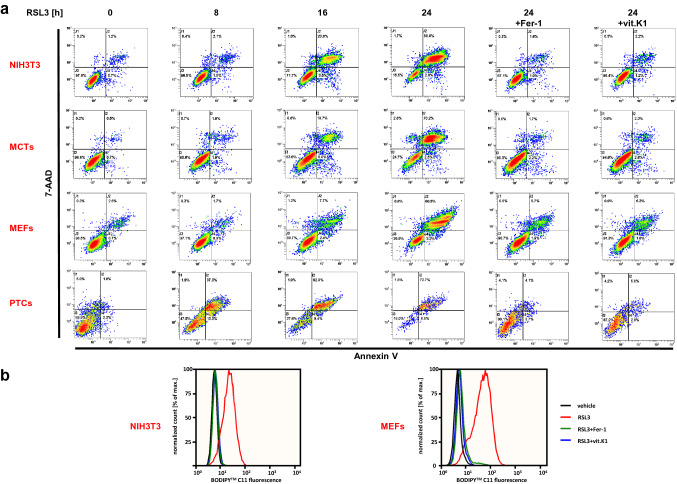
Vitamin K1 inhibits RSL3-induced ferroptosis in vitro. **a** Murine NIH3T3 cells, MCTs, MEFs, and PTCs were left untreated or stimulated at 37 °C for different durations with 5 µM RSL3 in the presence or absence of 1 µM ferrostatin-1 (Fer-1) or 10 µM vitamin K1 (vit.K1), as indicated. Cell death was quantified by FACS analysis using 7-amino-actinomycin D (7-AAD) and phosphatidylserine accessibility (Annexin V staining) as markers. **b** Examination of lipid ROS production preceding ferroptosis using BODIPY™ (581/591) C11. Indicated cells were treated at 37 °C for 4 h with 5 µM RSL3 (± 1 µM Fer-1 or 10 µM vit.K1). Overlay images show detection of accumulated ROS over time. A minimum of 10,000 cells were analyzed per condition. Notably, a shorter stimulation with RSL3 than in **a** was required for this assay, since ROS production in ferroptosis typically precedes triggering of cell death by several hours. Representative results of one of three independent experiments are shown

The protective effect of vitamin K1 against ferroptosis was reproducible in further immortalized cell lines as well as freshly isolated MEFs and PTCs (Fig. 2a). Thus, vitamin K1 is a reliable inhibitor of ferroptosis in both transformed cell lines and primary cells. Ferroptosis is characterized by the accumulation of toxic lipid peroxides in the plasma membrane, and this can be prevented by lipophilic antioxidant substances such as ferrostatin-1 [7, 38]. Using the lipid peroxidation sensor BODIPY™ 581/591 C11 [39, 40], we found that lipid peroxidation induced by RSL3 in immortalized as well as primary cells could be prevented by vitamin K1 to the same extent as by the canonical ferroptosis inhibitor ferrostatin-1 (Fig. 2b).

### Vitamin K1 can compensate for multiple intrinsic ferroptosis-inhibiting systems

**Fig. 3:**
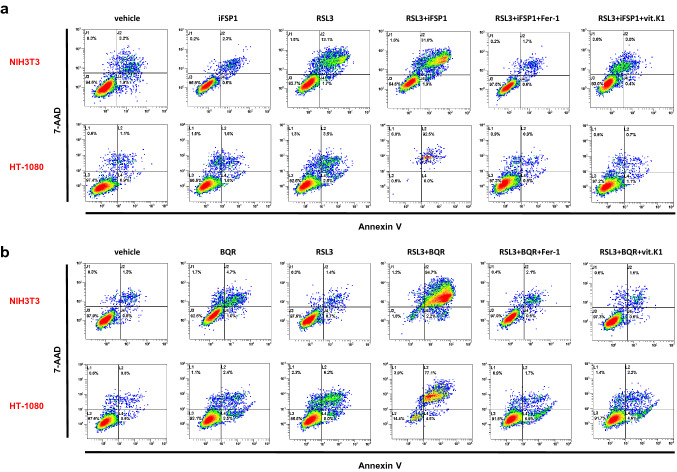
Vitamin K1 inhibits ferroptosis induced by inhibition of FSP1 or DHODH. NIH3T3 and HT-1080 cells were treated as indicated at 37 °C for 24 h with a sublethal dose of 0.1 µM (NIH3T3) or 1 µM RSL3 (HT-1080). The cell death-aggravating impact of **a** 10 µM iFSP1 or **b** 50 µM brequinar (BQR) is clearly evident under these conditions. Addition of 1 µM ferrostatin-1 (Fer-1) or 10 µM vitamin K1 (vit.K1) demonstrates that cell death perpetuated by iFSP1 or BQR is based on preventable ferroptosis. Cell death was quantified by FACS analysis using 7-amino-actinomycin D (7-AAD) and phosphatidylserine accessibility (Annexin V staining) as markers. FACS dot plots of one representative experiment are shown (*n* = 3 independent repeats)

Based on our findings described above (Fig. 1c), we assumed that the suppressive effect of vitamin K1 in ongoing ferroptosis was mechanistically associated with the presence and activity of GPX4. However, beside the GPX4 system, at least two other major cellular defense mechanisms are known to suppress ferroptosis: (1) ferroptosis suppressor protein 1 (FSP1) on the plasma membrane [11, 12], and (2) the mitochondrially located enzyme dihydroorotate dehydrogenase (DHODH) [13]. Using the FSP1 inhibitor iFSP1 and the DHODH inhibitor brequinar (BQR), respectively, we demonstrated that vitamin K1 can compensate for the impairment of each anti-ferroptotic defense mechanism with efficacy comparable to ferrostatin-1 (Fig. 3a and b).

### Phenprocoumon promotes ferroptosis in vitro

**Fig. 4:**
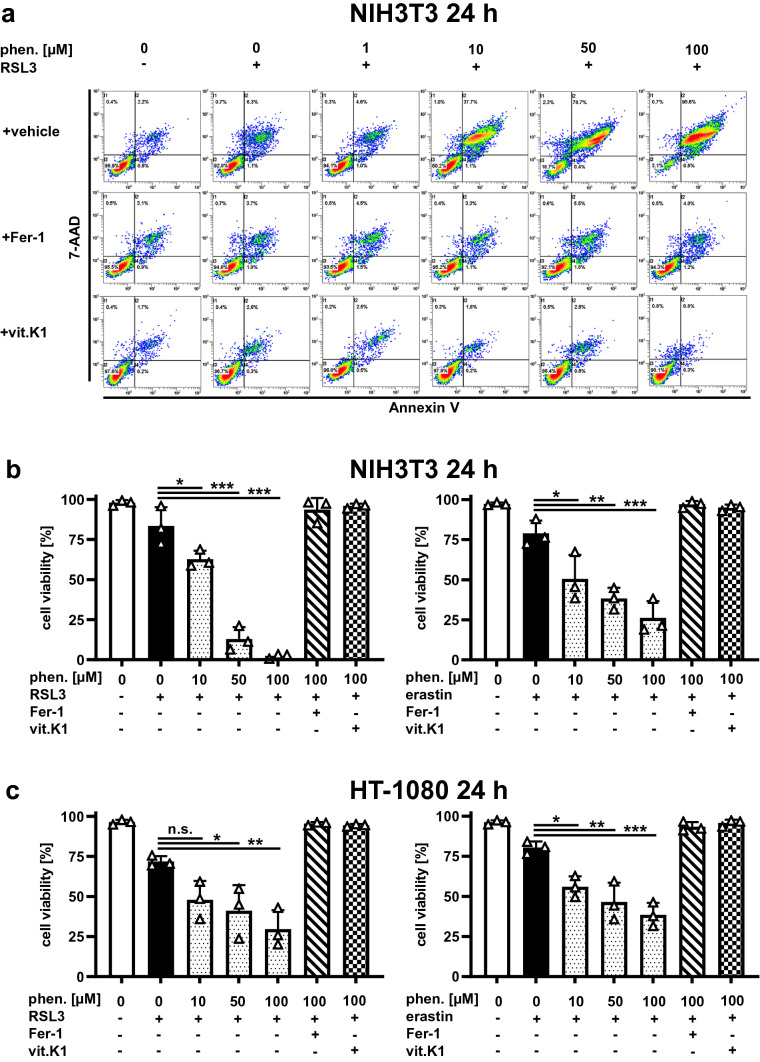
Phenprocoumon promotes ferroptosis in vitro*.* **a** Murine NIH3T3 cells were treated as indicated at 37 °C for 24 h with a sublethal dose of 0.1 µM RSL3. In the top row (vehicle), the aggravated cell death in the presence of increasing concentrations of phenprocoumon is clearly evident under these conditions. Addition of 1 µM ferrostatin-1 (Fer-1) or 10 µM vitamin K1 (vit.K1) illustrates that cell death mediated by phenprocoumon is based on preventable ferroptosis. FACS dot plots of one representative experiment are shown, the adjacent graph **b** presents the mean and SD of three independent experiments, and the identical effect of phenprocoumon using a sublethal dose of 0.2 µM erastin. **c** Phenprocoumon-induced facilitation of ferroptosis in vitro is species-independent, as confirmed using human HT-1080 cells. However, the human cells were treated as indicated at 37 °C for 24 h with a sublethal dose of 1 µM RSL3 and 2.5 µM erastin, respectively. Again, addition of 1 µM Fer-1 or 10 µM vit.K1 proved that the phenprocoumon-dependent effect was based on ferroptotic cell death. Cell death was quantified by FACS analysis using 7-amino-actinomycin D (7-AAD) and phosphatidylserine accessibility (Annexin V staining) as markers. Graphs show mean ± SD (*n* = 3 independent repeats)

Since the known physiological functions of vitamin K typically depend on vitamin K epoxide reductase (VKOR) [41, 42], we investigated whether the inhibitory effect of vitamin K1 on ferroptosis was also dependent on VKOR. For this purpose, we examined the effect of the vitamin K antagonist (VKA) phenprocoumon, a coumarin derivative and strong inhibitor of VKOR [43], on ferroptosis in vitro. When cells were incubated with phenprocoumon alone, we did not detect any kind of cell death, even after 24 h and with concentrations up to 100 μM (Supplemental Fig. 3). Surprisingly, however, increasing concentrations of phenprocoumon resulted in a significant enhancement of cell death under different ferroptosis conditions when induced with relatively weak stimuli. This effect was detectable in both RSL3- and erastin-induced ferroptosis and was consistent with observations in murine and human cells (Fig. 4a–c). Notably, the cell death additionally triggered by supplementation of phenprocoumon was completely reversed by both vitamin K1 and ferrostatin-1 (Fig. 4a–c). These results indicate that phenprocoumon aggravates ferroptosis induced by other stimuli, which is counteracted by vitamin K1 as well as the canonical ferroptosis inhibitor ferrostatin-1, whereas inhibition of VKOR alone does not induce ferroptosis.

### Vitamin K1 is protective in an in vivo model of renal IRI

**Fig. 5:**
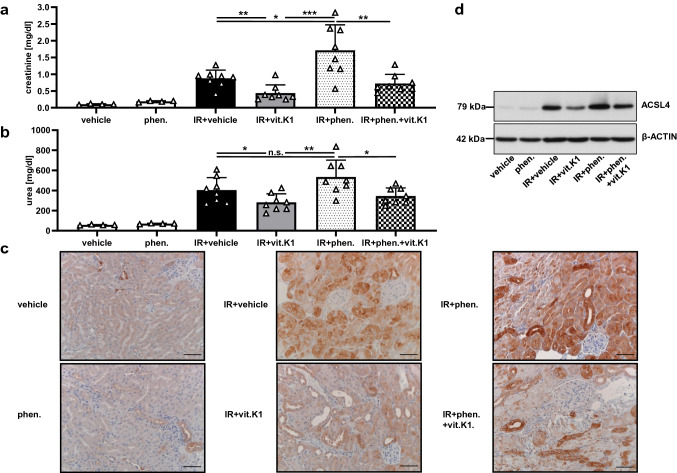
Vitamin K1 provides protection against renal ischemia–reperfusion injury, whereas phenprocoumon enhances the damaging effect. The significant therapeutic in vivo effect of vitamin K1 (vit.K1) and the detrimental effect of phenprocoumon (phen.) were evident under severe test conditions. All mice underwent 35 min of bilateral renal pedicle clamping followed by 48 h of reperfusion. Under these conditions, 15 min before ischemia, each mouse received a single intraperitoneal injection (total volume per mouse = 200 μl) of either PBS (vehicle), 4 mg phen./kg body weight, 25 mg vit.K1/kg body weight, or a combination as indicated (*n* = 4–8 mice per group as indicated). In this setting, we observed that the vehicle-treated mice in the IR group had significantly higher plasma levels of creatinine (**a**) and urea (**b**) than the vitamin K1-treated animals. In contrast, animals pretreated with phenprocoumon showed pronounced AKI, whereas phenprocoumon without IRI did not cause notable kidney function impairment. Remarkably, vitamin K1 when co-administered with phenprocoumon attenuated the extent of this phenprocoumon-mediated renal damage significantly. (**c**) Corresponding histological ACSL4 expression in the kidney samples of groups presented in (**a**) and (**b**). Increased expression of ACSL4 in this IR setting indicates an important role of ferroptosis in the complex pathology of acute tubular injury. ACSL4-positive tubuli and augmented ACSL4 expression can be seen in acute tubular injury (IR + vehicle group) and especially in the IR + phenprocoumon group, correlating with severe acute tubular damage. Normal renal parenchyma samples taken from the vehicle and the phenprocoumon groups served as controls. The protective effect of vitamin K1 in this scenario, associated with decreased ACSL4 expression, is evident in the appropriately labeled groups (scale bars = 50 µm). **d** Analogous expression levels of ACSL4 in whole-kidney lysates taken from the different groups presented in (**a**) and (**b**). The indicated samples were obtained after reperfusion. Equal amounts of protein (20 µg/lane) were resolved by SDS-PAGE, and expression of ACSL4 was detected by western blotting. The blot was stripped and re-probed with an antibody against β-actin as a loading control

We and others have previously demonstrated that ferroptosis plays a critical role in the pathogenesis of various forms of AKI, particularly in renal IRI [3–5, 44]. We, therefore, proceeded to investigate, whether vitamin K1 had a protective effect on AKI by inhibiting ferroptosis in vivo. For this purpose, we subjected mice on a C57BL/6 J background to renal IRI. Animals underwent 35 min of bilateral renal pedicle clamping followed by 48 h of reperfusion. After this time, ischemia–reperfusion (IR) animals treated only with vehicle displayed AKI as measured by elevated plasma levels of creatinine and urea. In contrast, animals pretreated with vitamin K1 showed significantly lower impairment of renal function (Fig. 5a and b). Consistent with the improved renal function parameters, animals in the IR+ vitamin K1 group showed significantly lower levels of tubular necrosis in comparison to the IR+ vehicle group (Supplemental Fig. 4a and b), which is thought to be mainly mediated by ferroptosis under renal IRI conditions [4, 5, 44]. In accordance with our previous work [5, 44], we observed a marked increase in expression of ACSL4 in kidneys following IRI, which was correlated with the severity of AKI. Treatment with vitamin K1 reduced tubular ACSL4 expression compared to the IR+ vehicle group, as revealed by immunohistochemistry (Fig. 5c) and western blots of whole kidney lysates (Fig. 5d).

### Phenprocoumon is detrimental in experimental AKI

Based on our in vitro data (Fig. 4), we hypothesized that phenprocoumon would aggravate ferroptotic cell death if it occurred in the context of other stimuli, such as renal IRI. Indeed, animals pretreated with phenprocoumon showed marked AKI and tubular necrosis in IRI, whereas phenprocoumon without IRI did not cause notable kidney function impairment in mice (Fig. 5a and b and Supplemental Fig. 4). Renal expression of ACSL4 as a marker of ferroptosis was further increased in the IR + phenprocoumon group compared to the other groups (Fig. 5c and d). Simultaneous additional treatment with vitamin K1 significantly preserved renal function parameters compared with the IR + phenprocoumon group (Fig. 5a and b). Renal ACSL4 expression was again reduced by concomitant vitamin K1 application in phenprocoumon-treated IR animals (Fig. 5c and d). None of the animals showed signs of excessive bleeding and hemoglobin levels between IR + vehicle and IR + phenprocoumon groups were indistinguishable (data not shown), ruling out exacerbated blood loss as a contributing factor to AKI.

In summary, our in vivo experiments revealed that (1) vitamin K1 inhibits ferroptosis in the course of AKI and thereby preserves kidney function, and (2) phenprocoumon aggravates ferroptosis during AKI, which can, for the most part, be alleviated by simultaneous administration of vitamin K1.

## Discussion

In the present study, we assessed the effectiveness of exogenously supplied vitamin K1 for inhibiting ferroptosis in vitro, and explored its therapeutic effect in a preclinical murine model of AKI. Furthermore, we investigated the role of the VKA phenprocoumon in aggravating ferroptosis in vitro and in AKI as well as its interplay with vitamin K1 in ferroptosis.

Our first major finding was that exogenous vitamin K1 efficiently inhibited ferroptosis induced by RSL3 or erastin in transformed murine and human cell lines, as well as in primary MEFs and PTCs. Interestingly, on one hand, we demonstrated that the suppressive effect of vitamin K1 during ferroptosis is associated with restoring the presence and activity of GPX4 (Fig. 1c). On the other hand, we also revealed that vitamin K1 sufficiently prevents ferroptotic cell death induced by inhibition of FSP1 or DHODH (Fig. 3). FSP1 is recruited in a GPX4-independent manner to the plasma membrane during ferroptosis, where it functions as an oxidoreductase that reduces coenzyme Q10 [11, 12], whereas DHODH prevents mitochondrial lipid peroxidation and ferroptosis [13]. Thus, vitamin K1 appears to efficiently act as a ferroptosis inhibitor independent of subcellular localization; it functions in the plasma membrane, cytosol, and mitochondria. It is worth mentioning that our studies did not reveal any difference in the mechanism of action between vitamin K1 and the lipophilic antioxidant ferrostatin-1, a canonical inhibitor of ferroptosis.

To date, only two modes of action of pharmacological cell death inhibitors have been suggested for all types of ferroptosis. To efficiently inhibit ferroptosis regardless of the initial trigger, compounds must either be strong iron chelators, such as deferoxamine, or potent lipophilic radical scavengers, so-called radical-trapping antioxidants (RTAs), such as ferrostatin-1 [7, 16, 17, 45]. Most likely, the inhibitory effect of vitamin K1 on ferroptosis is due to its property as an RTA, because vitamin K1 is not known to function as a chelator, but it is an effective reducing agent for lipid hydroperoxides in membrane vesicles [25, 26]. In addition, vitamin K may play an important role as a cofactor in redox systems at the plasma membrane [46] or mitochondria [47], and it may protect neurons and oligodendrocytes against oxidative cell death [28, 29]. In this context, we demonstrated complete prevention of ferroptotic lipid peroxidation using vitamin K1. Our study, therefore, provides further important evidence that vitamin K may be of physiological significance as an antioxidant agent, especially for ferroptosis.

Consistent with our in vitro findings, we demonstrated that vitamin K1 is protective in a murine model of renal IRI. In addition to significantly improved renal function, kidneys of mice pretreated with vitamin K1 showed a substantial reduction in tubular necrosis. Genetic studies and preclinical disease models have clearly established that ferroptosis plays a critical role in acute tubular necrosis, particularly when it is induced by IRI [3–5]. The enzyme ACSL4 is involved in this process as a critical regulator that allows ferroptosis to proceed by supplying long unsaturated and easily oxidizable ω6 fatty acids to the plasma membrane [35, 48, 49]. This enzyme has also been established as a biomarker of ferroptosis, including in AKI [5, 36, 44]. Consistent with enhanced tubular necrosis—and thus enhanced intrarenal ferroptosis—we found that renal expression of ACSL4 was markedly enhanced by IRI and reduced by vitamin K1. In summary, we revealed for the first time that vitamin K1 is an effective inhibitor of ferroptosis in vitro and in vivo. Our results pave the way for investigating the effectiveness of vitamin K1 in the therapy of AKI in humans. Translation to clinical applications may be relatively straightforward because vitamin K1 is already approved for use in humans, and it offers several advantages over other commonly studied ferroptosis inhibitors that are either not FDA-approved (e.g., ferrostatins or liproxstatin-1), have poor plasma stability [50], or have a worse safety profile than vitamin K1 (e.g., iron chelators or vitamin E) [51, 52].

In mammals, vitamin K is a crucial coenzyme in the process of γ-carboxylation of amino acid residues, a rather rare posttranslational modification, which is required for the functioning of specific proteins necessary for blood coagulation as well as calcium and bone metabolism [41]. Furthermore, vitamin K metabolism is thought to play a critical role in cardiovascular calcification and associated complications, particularly in patients with chronic kidney disease [22, 53]. A protein centrally involved in these vitamin K-dependent processes is VKOR, which is targeted by VKAs, certain derivatives of coumarins that are frequently used for anticoagulation in the clinic [42, 43]. Following our findings on the protective effect of exogenous vitamin K1 in ferroptosis, we further demonstrated that ferroptotic cell death is significantly exacerbated in the presence of the clinically established VKA phenprocoumon in vitro. To our knowledge, this is the first evidence showing that endogenous vitamin K1 and VKOR may play a previously unknown protective role in ferroptosis.

In retrospect, the ferroptosis-promoting effect of VKAs could also explain some interesting past observations, especially in oncology. Since the 1960s, coumarin derivatives have been known to prevent the occurrence of cancer metastases independently of anticoagulation, but this is dependent on vitamin K [54–56]. Nowadays, we know that both carcinogenesis and metastasis are intimately associated with ferroptosis [57, 58]. These mechanistic considerations are supported by clinical data showing that long-term therapy with VKAs reduces cancer incidence and anticoagulation with warfarin improves survival in certain cancers [59, 60]. Induction of ferroptosis has now been clearly linked to cancer therapy. In particular, adjunction of classical chemotherapeutic agents, but also new immunotherapies with ferroptosis inducers, are very promising [61, 62]. Based on our current study, it seems desirable to further investigate the importance of the vitamin K cycle and a possible therapeutic use of VKAs in the context of oncological ferroptosis induction.

Although we could not shed further light on the detailed mechanism by which the vitamin K cycle and VKOR contribute to the cellular response to ferroptotic stimuli, we herein present in vivo findings of potential clinical relevance in nephrology. The VKA phenprocoumon aggravates impairment of kidney function in a preclinical model of AKI by driving ferroptosis. This prompts the pertinent question whether patients treated with VKAs during AKI have a worse outcome. Unfortunately, prospective controlled clinical studies specifically investigating this issue have not been reported to date. Nevertheless, several retrospective observational studies including tens of thousands of patients support the assumption that VKAs may influence the occurrence of AKI and worsen renal outcomes [63–65]. A link between VKAs and kidney failure has also been postulated under the name warfarin- or anticoagulant-related nephropathy [66, 67]. Mechanistically, this has been associated primarily with overanticoagulation, glomerular hemorrhage, and obstructive red blood cell casts, although this probably affects only a minority of patients and is not conclusively reproducible in animal models [68–71]. Evidence has been provided previously in animal models that oxidative stress may be involved in the development of warfarin-related nephropathy [72]. Based on our current results, we provide a possible mechanism by which VKAs may contribute to renal injury apart from increased bleeding tendency through enhanced induction of ferroptosis. Our findings are of particular clinical relevance in this setting because we demonstrated that concomitant administration of vitamin K1 can reverse kidney injury aggravated by phenprocoumon via the anti-ferroptotic properties of vitamin K1. In particular, since vitamin K1 is nontoxic, this could provide a rationale for administering vitamin K1 to patients anticoagulated with VKAs in an AKI setting. Further clinical studies are needed to explore this hypothesis.

In summary, we identified vitamin K1 as a potent inhibitor of ferroptosis, and hence, it represents a potential drug for the treatment of pathological cell death processes during AKI in humans. Conversely, vitamin K antagonists promote ferroptosis during AKI, which could explain previous reports of new or increased AKI and renal impairment during VKA-based therapy. In this regard, vitamin K1 is a potent antidote against ferroptosis promoted by VKAs. The role of the vitamin K cycle in ferroptosis needs further investigation and may hold enormous therapeutic potential for a multitude of diseases associated with this mode of cell death.

### Supplementary Information

Below is the link to the electronic supplementary material.

Supplementary file1 (PDF 781 kb)

## Data Availability

All data and information regarding material will be available upon reasonable request.
