# Supplementary material for: Vitamin K1 inhibits ferroptosis and counteracts a detrimental effect of phenprocoumon in experimental acute kidney injury
Source: Cell Mol Life Sci. 2022 Jun 28;79(7):387. doi: 10.1007/s00018-022-04416-w (PMC9239973; doi:10.1007/s00018-022-04416-w)
Supplement: Supplementary file 1 — Supplementary file1 (PDF 781 kb) [file 18_2022_4416_MOESM1_ESM.pdf]

Supplemental Figures

Vitamin K1 inhibits ferroptosis and counteracts a detrimental effect of phenprocoumon in experimental acute kidney injury

Benedikt Kolbrink[1] · Friedrich Alexander von Samson-Himmelstjerna[1] · Maja Lucia Messtorff[1] · Theresa Riebeling[1] · Raphael Nische[1] · Jessica Schmitz[2] · Jan Hinrich Bräsen[2] · Ulrich Kunzendorf[1] · Stefan Krautwald[1]

[1]Department of Nephrology and Hypertension, University Hospital Schleswig-Holstein, 24105 Kiel, Germany

[2]Nephropathology Unit, Institute of Pathology, University of Hannover, 30625 Hannover, Germany

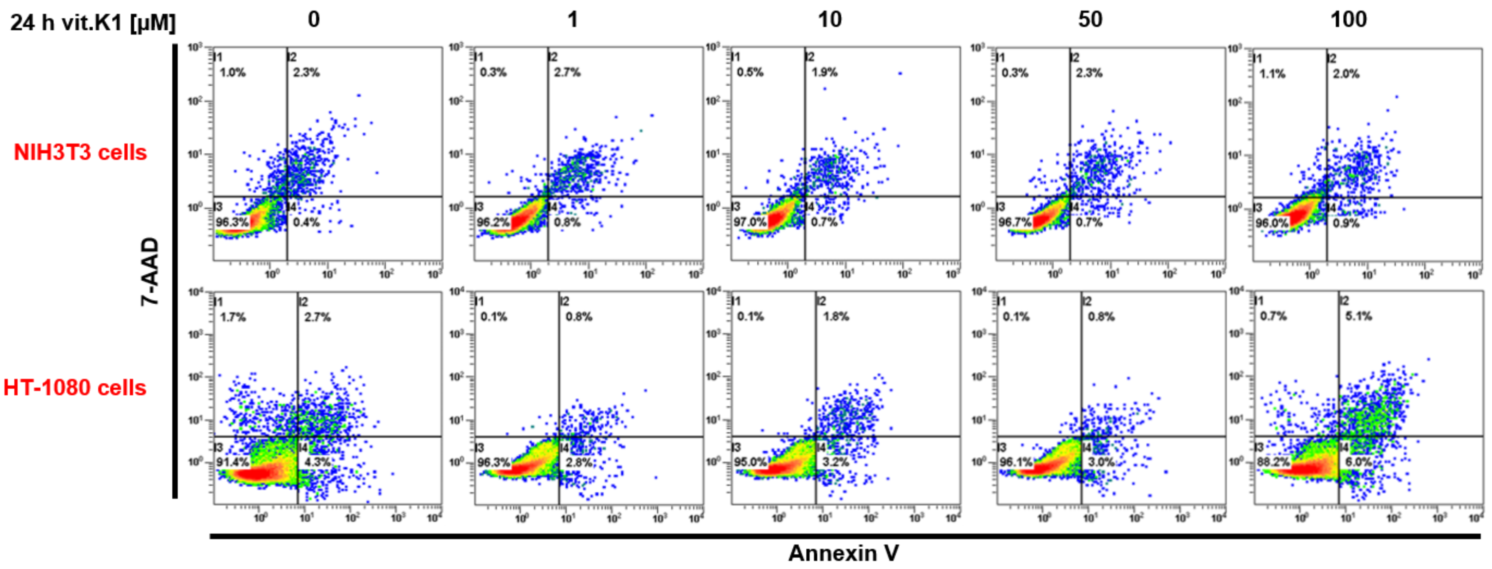

Supplemental Figure 1. Vitamin K1 is not cytotoxic by itself. Murine NIH3T3 as well as human HT-1080 cells were each treated at 37°C for 24 h in the mere presence of increasing concentrations (up to 100 µM) of vitamin K1 (vit.K1). Cell death was analyzed by FACS analysis using 7-amino-actinomycin D (7-AAD) and phosphatidylserine accessibility (Annexin V staining) as markers. FACS dot plots of one representative experiment are shown; n = 3 independent repeats.

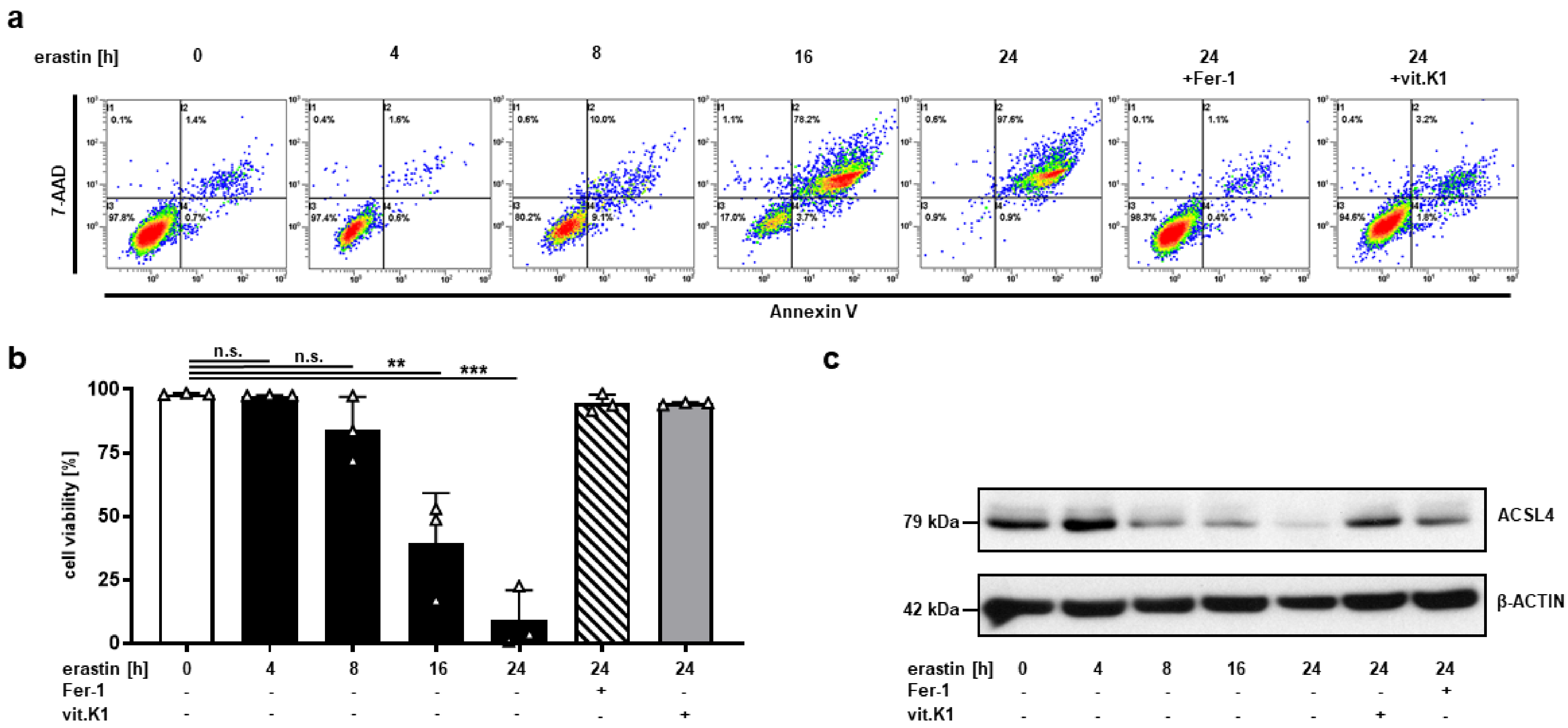

Supplemental Figure 2. Vitamin K1 inhibits erastin-induced ferroptosis *in vitro*. Murine NIH3T3 cells were left untreated or were stimulated at 37°C for different durations with 2.5 µM erastin in the presence or absence of 1 µM ferrostatin (Fer-1) or 10 µM vitamin K1 (vit.K1), as indicated. Cell death was quantified by FACS analysis using 7-amino-actinomycin D (7-AAD) and phosphatidylserine accessibility (Annexin V staining) as markers. **(a)** FACS dot plots of one representative experiment are shown, the adjacent graph **(b)** presents the mean and standard deviation of three independent experiments. One representative corresponding immunoblot is shown in **(c)**. Equal amounts of protein (20 µg/lane) were resolved by SDS-PAGE, and expression of ACSL4 was detected by western blotting. The blot was stripped and re-probed with an antibody against β-actin as a loading control.

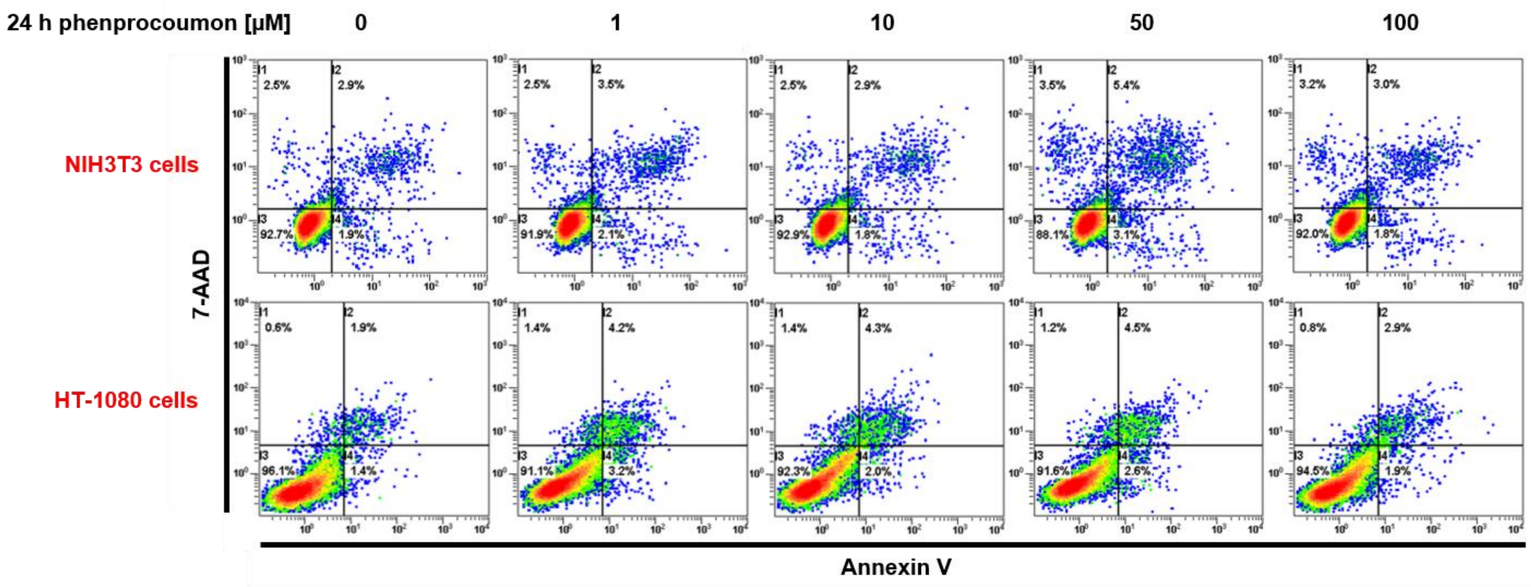

Supplemental Figure 3. The vitamin K antagonist phenprocoumon is not cytotoxic by itself. Murine NIH3T3 as well as human HT-1080 cells were each treated at 37°C for 24 h in the mere presence of increasing concentrations (up to 100 µM) of phenprocoumon. Cell death was analyzed by FACS analysis using 7-amino-actinomycin D (7-AAD) and phosphatidylserine accessibility (Annexin V staining) as markers. FACS dot plots of one representative experiment are shown; n = 3 independent repeats.

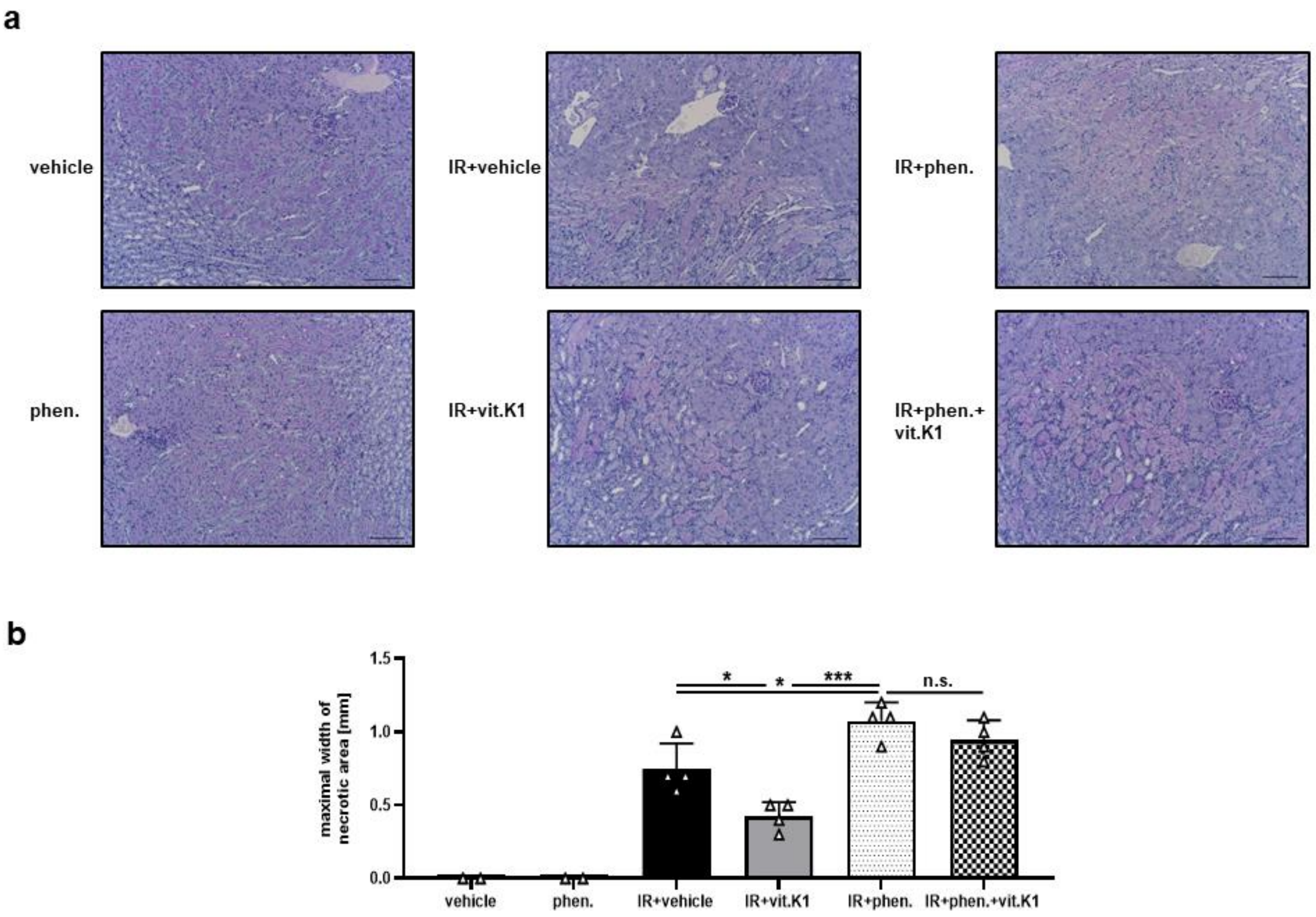
a
vehicle
IR+vehicle
IR+phen.
phen.
IR+vit.K1
IR+phen.+
vit.K1
b
maximal width of
necrotic area [mm]
1.5
1.0
0.5
0.0
*
*
***
n.s.
vehicle
phen.
IR+vehicle
IR+vit.K1
IR+phen.
IR+phen.+vit.K1

Supplemental Figure 4. **Vitamin K1 provides protection from ischemia-reperfusion damage, whereas phenprocoumon enhances the damaging effect. (a)** Representative histological sections of murine kidneys presented in Figure 5 after staining with periodic acid-Schiff (PAS). The significant therapeutic *in vivo* effect of vitamin K1 and the detrimental effect of phenprocoumon was evident under severe test conditions. As described before, all the mice underwent 35 min of bilateral renal pedicle clamping followed by 48 h of reperfusion. 15 min before ischemia the mice received each a single intr aperitoneal injection (total volume per mouse = 200 µl) of either PBS (vehicle), 4 mg phenprocoumon (phen.)/kg body weight, 25 mg vitamin K1 (vit.K1)/kg body weight or a combination as indicated. Animals pretreated with phenprocoumon showed very pronounced AKI and tubular necrosis, whereas phenprocoumon without IRI did not cause notable impairment of kidney structure. Remarkably, vitamin K1 when administered in IRI alone or when co-administrated with phenprocoumon was able to attenuate the extent of kidney injury in a significant manner. Depicted are representative enlarged sections of each group after the reperfusion. Scale bars = 100 µM. **(b)** Maximal size of necrotic area (tubular damage) was graded in blinded samples by an experienced renal pathologist.
